# Determinant Factors of Self‐Care Deficit in People With Chronic Wounds: A Scoping Review

**DOI:** 10.1111/jan.70628

**Published:** 2026-04-30

**Authors:** Janislei Soares Dantas, Yan Lincoln Mamede Gomes, Ana Clara de Macedo Farias Ramos, Selene Cordeiro Vasconcelos, Maria Eliane Moreira Freire

**Affiliations:** ^1^ Postgraduate Nursing Program/Federal University of Paraíba (PPGENF/UFPB) Lauro Wanderley University Hospital‐Brazilian Company of Hospital Services (HULW/EBSERH), Federal University of Paraíba (UFPB) João Pessoa Paraíba Brazil; ^2^ Clincal Nursing Departament Federal University of Paraíba (UFPB) João Pessoa Paraíba Brazil; ^3^ Postgraduate Nursing Program/Federal University of Paraíba (PPGENF/UFPB) Federal University of Paraiba João Pessoa Paraíba Brazil

**Keywords:** chronic wounds, nursing, nursing theory, self care, social determinants of health, wound care

## Abstract

**Aims:**

To identify and synthesise the scientific evidence on determinants of self‐care deficits in people with chronic wounds.

**Design:**

Scoping review based on the Joanna Briggs Institute guidelines.

**Methods:**

This scoping review was conducted by a review team responsible for study screening, data extraction and synthesis. The categorisation of findings was grounded in Orem's Self‐Care Deficit Theory and operationalised using the COM‐B model and the Theoretical Domains Framework.

**Data Sources:**

A comprehensive search, without date or language restrictions, was conducted in Web of Science, MEDLINE (PubMed), Cochrane Library, Scopus (Elsevier), SciELO, Ovid and EMBASE (Elsevier), from November 2023 to April 2024.

**Results:**

Of the 3076 records identified, 22 studies were included. Most were cross‐sectional studies, with sample sizes ranging from seven to 1085 participants and a mean age of 61.45 years, predominantly involving people with venous leg ulcers and diabetic foot ulcers. Findings were synthesised into four categories: clinical, socioeconomic, behavioural and health system‐related factors, indicating that multiple interrelated determinants influence engagement in self‐care and contribute to persistent self‐care deficits.

**Conclusion:**

The findings demonstrate the vulnerability of people living with chronic wounds, arising from the complex interaction of clinical, psychosocial, socioeconomic and health service‐related factors that determine self‐care deficits. These deficits compromise wound healing, prolong treatment and negatively affect quality of life, underscoring the need for person‐centred approaches to care.

**Implications for the Profession and/or Patient Care:**

This study reinforces the need for integrated and sustainable models of care grounded in person‐centred practice, aimed at strengthening self‐care capacities, enhancing clinical practice and informing more equitable health policies.

**Impact:**

This review highlights the multifactorial nature of self‐care deficits in people with chronic wounds. Nursing plays a key role in identifying barriers and strengthening capacities through Orem's supportive–educative system. Person‐centred interventions improve adherence and continuity of care.

**Reporting Method:**

This review follows the PRISMA guidelines for reporting.

**Patient or Public Contribution:**

This study did not include patient or public involvement in its design, conduct, or reporting.

## Contribution to the Wider Global Clinical Community

The study reinforces the central role of nursing in strengthening self‐care capacities and improving equity and continuity in chronic wound care across diverse global settings.

## 1 Introduction

Wounds are often initially managed by individuals themselves, their informal care networks or non‐specialised primary healthcare services. A major concern in wound management is the high risk of chronicity, which is associated with an increased likelihood of complications, functional impairment, physical and emotional distress, reduced quality of life and the need for complex and costly therapies for both individuals and health systems (Eckert et al. 2023; Dantas et al. 2022; Beeckman et al. 2024).

A wound is defined as any disruption of the structural and functional integrity of the skin. Chronic wounds, in contrast, are characterised by delayed or impaired healing due to factors such as unfavourable anatomical location, clinical, psychological or socioeconomic conditions, or failure to respond to evidence‐based care (Murphy et al. 2022). Importantly, wounds should not be understood solely as isolated skin lesions. This broader perspective shifts the focus from the wound itself to the person living with the condition, emphasising the need for care approaches that address physical, emotional, social and spiritual dimensions.

Contemporary data indicate that wound prevalence ranges from 1% to 5% in developed countries (Berenguer‐Pérez et al. 2024). In the United States, chronic wounds affect approximately 16.4% of Medicare beneficiaries (Eckert et al. 2023). In Spain, the cumulative annual prevalence of chronic wounds was 88.7 per 10,000 inhabitants in 2017 (Díaz‐Herrera et al. 2025). In Brazil, although systematic national data are lacking, it is estimated that around 3% of the population lives with some type of skin lesion (Melo et al. 2020). Globally, the prevalence of chronic wounds is expected to increase in the coming decades, driven by population ageing and the growing burden of chronic diseases (Díaz‐Herrera et al. 2025).

Despite technological advances in topical therapies and wound‐healing strategies, significant gaps remain in the care provided to people with chronic wounds, particularly in relation to psychological, social and educational dimensions (Tatarusanu et al. 2023). These aspects are central to promoting person‐centred care and strengthening individuals' self‐care capacities, thereby supporting therapeutic continuity and empowerment in health recovery (Sengul et al. 2022). However, national and international evidence indicates that wound care remains predominantly biomedical, focused on local wound treatment, fragmented across levels of care and frequently disconnected from comprehensive, person‐centred approaches (Gethin et al. 2020; Marques et al. 2024).

In response to these limitations, recent studies have highlighted the need for new paradigms in wound care, including sociogenomic approaches. Evidence suggests that social determinants, living environments and psychosocial stress influence wound healing by modulating pain perception, infection risk, inflammatory and hormonal responses, as well as gene expression during tissue repair (Sen and Roy 2019). This understanding is reinforced by research identifying multiple determinants of wound healing, including biological and systemic conditions, social environment, nutrition, socioeconomic status, educational level, social isolation and situational stressors (Fayne et al. 2020; Matar et al. 2023; Hajj et al. 2024). Collectively, these findings support the need for multifaceted, evidence‐based approaches that integrate clinical, social and contextual factors to optimise wound outcomes (Gethin et al. 2020; Eriksson et al. 2022).

Within this context, people living with chronic wounds are increasingly involved in managing their own care, including wound cleansing, dressing changes and the use of alternative therapies. Self‐care has therefore become a fundamental component of long‐term wound management, with its relevance further intensified during the COVID‐19 pandemic (Kapp et al. 2022).

Self‐care, as conceptualised by Dorothea Elizabeth Orem, refers to purposeful activities initiated and performed by individuals to maintain life, health and well‐being. Self‐care capacity is influenced by fundamental conditioning factors—both internal and external to the individual—including age, health status, sociocultural orientation, living conditions, environmental factors and access to resources (Orem 1995). When the quantity or quality of self‐care is insufficient to meet therapeutic demands, a self‐care deficit occurs, activating the need for nursing systems of care (Neves et al. 2021).

Considering the interrelationships between self‐care, social determinants and sociogenomic processes offers opportunities to improve clinical outcomes, reduce the economic burden associated with chronic wounds and enhance quality of life through multidisciplinary care, shared decision‐making and comprehensive treatment planning (Sen 2025). Nevertheless, the academic literature addressing the specific determinants of self‐care deficits in people living with chronic wounds remains limited.

Therefore, this scoping review aims to identify and synthesise the scientific evidence on determinants of self‐care deficits in people living with chronic wounds. A deeper understanding of these determinants is essential to support healthcare professionals in planning person‐centred care aligned with individuals' lived realities.

## 2 The Review

This scoping review was conducted in accordance with the Joanna Briggs Institute (JBI) methodology (Peters et al. 2024). Scoping reviews are a form of evidence synthesis designed to identify and map the breadth of available literature on a given topic, concept or research question within or across contexts. They can inform clinical practice guidelines and policy development, serve as a precursor to systematic reviews, or guide future primary research (Pollock et al. 2024).

A preliminary search of databases, including MEDLINE and the Cochrane Database of Systematic Reviews, identified no current or ongoing reviews addressing this topic.

## 3 Aims

This review aimed to identify and synthesise the scientific evidence on determinants of self‐care deficits in people living with chronic wounds. The review question was: What scientific evidence is available in the literature regarding determinants of self‐care deficits in people with chronic wounds?

## 4 Methods

### 4.1 Design

The protocol for this scoping review was developed in accordance with the JBI Manual for Evidence Synthesis (Peters et al. 2024). Reporting followed the Preferred Reporting Items for Systematic Reviews and Meta‐Analyses extension for Scoping Reviews (PRISMA‐ScR) (Tricco et al. 2018). Recommendations for data extraction, analysis and presentation were informed by Pollock et al. (2022).

The review protocol was registered in the Open Science Framework (OSF) on 12 August 2024 (DOI: https://doi.org/10.17605/OSF.IO/EC9WZ).

### 4.2 Search Methods

An initial limited search of MEDLINE (PubMed) was undertaken using key terms related to the Population, Concept and Context (PCC) framework to identify relevant studies. Keywords and index terms identified in the titles and abstracts of retrieved articles were then used to develop a comprehensive search strategy tailored to each database to maximise sensitivity and precision, as detailed in Table 1.

**TABLE 1:** Inclusion and exclusion criteria (PCC).

Structure	Inclusion criteria	Exclusion criteria	Key terms
Population	People with chronic wounds, adults and/or elderly people of both sexes, with no upper age limit. Primary research studies, available in full	People with neoplastic wounds, due to the specificity of their scar physiology	“Wounds and injuries” “Wounds” “Chronic wounds” “wounds hard to heal” “ulcer” “skin ulcer” “chronic ulcers” “leg ulcer” “venous ulcer” “foot ulcer” “diabetic foot” “pressure ulcer”
Concept	Factors and/or determinants of deficits in self‐care and self‐management of the wound. D. Orem's Self‐Care Theory	Articles that do not address the focus of the research question	Self‐care Self‐management Self‐treatment Patient participation
Context	People in any healthcare setting where people with chronic wounds are cared for	Methodological studies, scientific articles in abstract format	Healthcare environment

*Source:* Own author.

The following databases were searched: Web of Science, MEDLINE (PubMed), Cochrane Library, Scopus (Elsevier), SciELO, Ovid and Embase (Elsevier). Searches covered publications from November 2023 to April 2024. The full search strategy is presented in Appendix 1. Ethical approval was not required, as this review was based exclusively on previously published data.

### 4.3 Inclusion and Exclusion Criteria

Eligibility criteria were defined using the Population, Concept and Context (PCC) framework (Peters et al. 2024). Population included people of any age and sex living with chronic wounds of any aetiology; the Concept was self‐care deficit; and the Context comprised all care settings, with no restriction on level of care. Table 1 details the eligibility criteria and the controlled vocabulary used to develop the search strategy. No restrictions were applied regarding language, publication date, geographic location or cultural context.

### 4.4 Search Outcome

The Rayyan‐Intelligent Systematic Review (Rayyan) software was used to reference manager. A structured, multi‐stage process was undertaken for study selection and data analysis. Titles and abstracts were independently screened by two reviewers in a blinded manner; at this stage, the rate of selection disagreement was below 10%. Reference lists of included studies were manually searched to identify additional relevant articles. Any disagreements were resolved through discussion with a third reviewer.

### 4.5 Quality Appraisal

Although formal quality appraisal is not mandatory in scoping reviews, the hierarchy of evidence was classified descriptively according to Melnyk and Fineout‐Overholt (2005). This classification was used solely to provide an overview of the types and levels of evidence available and did not imply methodological quality assessment or strength of recommendations, in line with JBI and PRISMA‐ScR guidance.

### 4.6 Data Abstraction and Synthesis

Full‐text articles were assessed by the review team using a structured process. Two reviewers independently examined the full texts in a blinded manner. Disagreements were resolved by consensus, with the involvement of a third reviewer, who also checked the consistency of the extracted data. Data extraction was performed using a predesigned form adapted from Peters et al. (2024) to summarise the key methodological and descriptive characteristics of each included study.

The categorisation of findings was grounded in Orem's Self‐Care Deficit Theory (Orem 1971; Orem 1995), and the analysis of determinants of self‐care deficits was operationalised using the COM‐B model (Capability, Opportunity, Motivation–Behaviour) and the Theoretical Domains Framework (TDF). Even when studies did not explicitly use the term self‐care deficit, an inferential approach anchored in Orem's theoretical constructs—integrated with COM‐B and TDF behavioural domains—was applied.

Accordingly, evidence of self‐care deficit included functional, cognitive or emotional limitations; socioeconomic and contextual barriers; inadequate health behaviours and motivational factors; and difficulties related to treatment adherence and health system support.

Following consolidation of the final analytical framework, coding was validated by a fourth reviewer to ensure analytical consistency, respect conceptual heterogeneity across studies and maintain fidelity to the adopted theoretical framework.

## 5 Results

### 5.1 Selection of Sources of Evidence

The database searches identified 3076 records. After duplicate removal and title and abstract screening, 39 full‐text articles were assessed for eligibility, of which 18 met the inclusion criteria. An additional four other relevant studies were identified through manual analysis of the reference lists of the included articles and also met the eligibility criteria. In total, 22 studies were included in the final sample. Figure 1 illustrates the flow of studies during the selection process.

**FIGURE 1:**
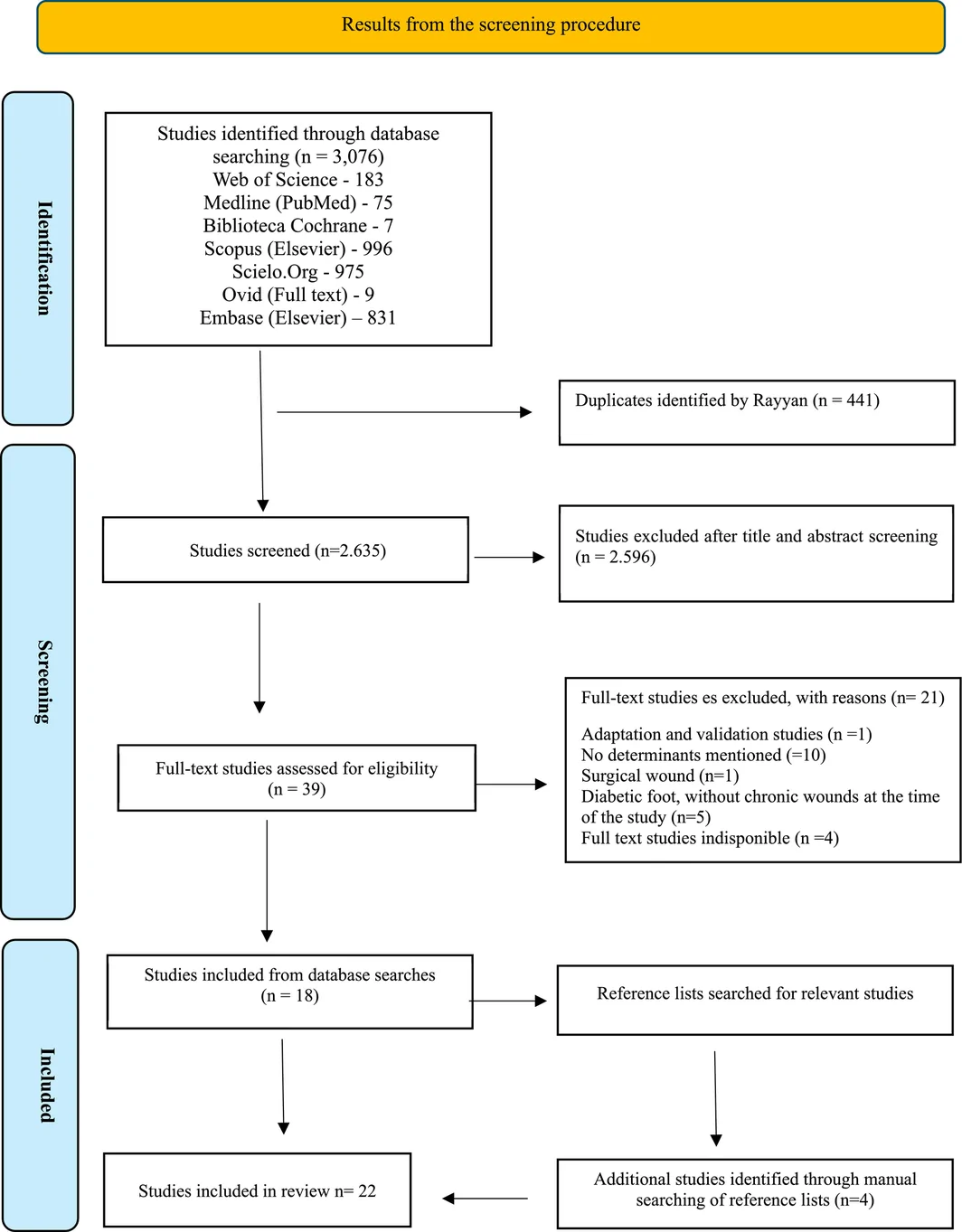
PRISMA flowchart.

### 5.2 Characteristics of Eligible Studies

Regarding methodological design, 16 studies (73%) were descriptive cross‐sectional studies, three (14%) were reviews, one (4%) was a cohort study, one (4%) was longitudinal and one (4%) was quasi‐experimental. Most studies were classified as level VI evidence. The studies were conducted across different countries and were predominantly published in peer‐reviewed scientific journals.

The combined sample represented 3063 people living with chronic wounds, with venous leg ulcers and diabetic foot ulcers being the most frequently reported wound types. Sample sizes ranged from seven to 1085 participants, with a mean age of 61.45 years (SD ±12.04). Across studies, men were slightly more prevalent than women. Detailed characteristics and coding of the included studies are presented in Table 2.

**TABLE 2:** Characterisation and synthesis of the studies included in the review (*n* = 22).

First Author/Year/País/Journal	Title	Objective	Study design/Level of evidence	Study population	Deficits found	Determinants of self‐care deficit
Jones et al. (2003) USA *Archives of Physical Medicine and Rehabilitation*	Use of behavioural contingencies to promote prevention of recurrent pressure ulcers	To determine the effectiveness of monetary rewards to prevent or reduce the severity of pressure ulcers in patients with spinal cord injury	Almost experimental Quantitative analysis Level 3	12 patients with severe pressure ulcers	Drug abuse, obesity, resistance to recommendations, chronic pain, depression, psychiatric impairment	Clinical Psychosocial Cognitive
Hopkins et al. (2006) UK *Journal of Advanced Nursing*	Patient stories of living with a pressure ulcer	To report experiences of elderly people with pressure ulcers	Cross‐sectional Qualitative analysis Level 6	8 patients with pressure ulcers Grade 3 or 4	Pain, comorbidities	Clinical
Gonzalez et al. (2010) USA *Diabetologia*	Depression predicts first but not recurrent diabetic foot ulcers	To examine the relationship between depression and diabetic foot ulcer development	Cross‐sectional Quantitative analysis Level 6	333 patients with diabetic neuropathy	Depression	Psycho‐emotional
Abu‐Qamar (2014) Jordan *Journal of Wound Care*	Knowledge and practice of foot self‐care among Jordanians with diabetes: An interview‐based survey study	To analyse the knowledge and practice of foot care among Jordanian patients with diabetes	Cross‐sectional Quantitative analysis Level 6	1085 people with diabetes‐related ulcers	Knowledge and practice of care for compromised feet	Cognitive
Vedhara et al. (2014) UK *Diabetes Research and Clinical Practice*	Illness beliefs predict self‐care behaviours in patients with diabetic foot ulcers: A prospective study	Examine the relationship between beliefs and self‐care	Cross‐sectional Quantitative analysis Level 6	169 patients with diabetic foot ulcers	Cultural beliefs, age, ulcer severity	Psycho‐emotional Clinical
Silva et al. (2016) Brazil *Estima—Brazilian Journal of Enterostomal Therapy*	The Self‐care Experience of Women Living with Chronic Venous Leg Ulcer	Understand the self‐care experience of women living with ulcers chronic venous	Cross‐sectional Qualitative analysis Level 6	7 women with venous ulcer	Lack of awareness of the person about their health condition, beliefs that interfere with food choices, pain and comorbidities	Cognitive Clinical
Garcia et al. (2018) Brazil *Revista Gaúcha de Enfermagem*	Perception of users on self‐care of lower leg ulcers	To know the user's perception of ulcer self‐care in the lower limbs.	Cross‐sectional Quantitative analysis Level 6	10 people with chronic wounds	Lack of skills to care for the injury, lack of personal motivation, inappropriate lifestyles, lack of support from professionals and the health network	Socioeconomic Cognitive Health system
Kapp et al. (2018) Australia *Journal of Clinical Nursing*	The quality of life of people who have chronic wounds and who self‐treat	Investigate the effect of chronic wounds on the physical domains, emotional, social, lifestyle and financial quality of life in people who treat their own wounds	Cross‐sectional Qualitative analysis Level 6	25 people With chronic wounds of varying origin	Financial costs of treatment	Socioeconomic
Mohammad et al. (2018) Saudi Arabia *Open Access Macedonian Journal of Medical Sciences*	Evaluate the Effect of Education Interventions in the Prevention of Diabetic Foot Ulcers through Knowledge of the Disease and Self‐Care Practices in Saudi Arabia	To evaluate the effect of educational interventions on the prevention of diabetic foot ulcers by knowledge of the disease and self‐care practices	Almost experimental; Quantitative analysis Level 3	60 people with diabetic foot ulcer	Lack of access to financial resources, low levels of knowledge and education that interfere with the adoption of foot wound care practices	Cognitive Socioeconomic
Phillips et al. (2018) UK *Journal of Advanced Nursing*	A systematic review of qualitative research into people's experiences of living with venous leg ulcers	Systematically identify, evaluate, and synthesise qualitative research that examined health‐related symptoms and quality of life themes	Systematic review of qualitative research. Level 5	13 studies	Lack of clear and accessible guidance on your health condition	Cognitive
Zulec et al. (2019) Croatia *International Journal of Environmental Research and Public Health*	“Wounds Home Alone”—Why and How Venous Leg Ulcer Patients Self‐Treat Their Ulcer: A Qualitative Content Study	To investigate why and how patients with venous ulcers self‐treat their ulcers	Cross‐sectional Qualitative analysis Level 6	32 people with venous ulcer	Financial constraints, lack of healthcare resources, and little patient knowledge of their condition	Cognitive Socioeconomic
Lacerda et al. (2019) Brazil *Revista Brasileira de Enfermagem*	Self‐care deficits in women with leg ulcers and sickle cell disease	Identify self‐care deficits in women with leg ulcers and disease sickle‐shaped	Cross‐sectional Qualitative analysis Level 6	14 women with leg ulcers secondary to sickle cell disease	Impaired sociability, ineffective food intake for ulcer healing, chronic condition that affects mobility, recurrence of ulcers, chronic ischemia pain, integrity of the affected skin and outdated vaccination cards	Clinical Socioeconomic
Chin et al. (2019) Taiwan *Journal of Clinical Nursing*	Factors associated with foot ulcer self‐management behaviours among hospitalised patients with diabetes	Determine self‐management behaviours of related foot ulcers to diabetes and explore the factors associated with these behaviours.	Cross‐sectional Quantitative analysis Level 6	199 people with diabetic foot ulcer	Lack of awareness of the need for self‐management of the ulcer, lack of problem‐solving capacity on the part of the health service, difficulty in asking for time off work to go to the hospital	Cognitive Socioeconomic Health System
Costa et al. (2020) Canada *Advances in Nursing Science*	I Cannot Afford Off‐loading Boots: Perceptions of Socioeconomic Factors Influencing Engagement in Self‐management of Diabetic Foot Ulcer	To discover the factors that influence individuals' ability to engage in diabetic foot ulcer self‐care and to present a theoretical model that describes these factors and their outcomes	Cross‐sectional Qualitative analysis Level 6	30 patients with diabetic foot ulcer	Ageing; comorbidities, lack of socio‐family support; difficulty in accessing and resolving health services and financial resources	Psychoemotional Clinic Cognitive Health system
Fayfman et al. (2020) USA *Journal of Diabetes and Its Complications*	Barriers to diabetic foot care in a disadvantaged population: A qualitative assessment	Explore barriers to proper foot care in people with diabetic foot ulcer	Cross‐sectional Quantitative‐qualitative analysis Level 6	40 people with diabetic foot ulcer	Lack of motivation, high cost of treatment, mobility difficulties, difficulty in making timely appointments and contacting a provider to arrange care	Cognitive Socioeconomic Health System
Balet et al. (2021) France *International Angiology*	Limitations to self‐management of adjustable compression wraps in the elderly: results of a prospective cohort study	Determine the main factors that prevent the self‐application of compression bandage to the lower limbs	Cohort; Level 4	30 people with venous ulcer	Obesity, cognitive impairment, and low social status	Clinical Socioeconomic Cognitive
Costa et al. (2021) Canada *Canadian Journal of Diabetes*	Patients' Journey Toward Engagement in Self‐Management of Diabetic Foot Ulcer in Adults With Types 1 and 2 Diabetes: A Constructivist Grounded Theory Study	To develop the first theory that could explain the processes of involvement in the self‐management of adults with diabetic foot ulcer	Cross‐sectional Qualitative analysis Level 6	30 people with diabetic foot ulcer	Insufficient information from physicians and non‐individualised guidance from health professionals, lack of understanding of the proper management of their ulceration	Cognitive Health system
Negash et al. (2022) Ethiopia *Journal of International Medical Research*	Prevalences of diabetic foot ulcer and foot self‐care practice, and associated factors in adult patients with diabetes in south‐east Ethiopia	To assess the prevalence of foot ulcers and foot self‐care practices and identify associated factors	Cross‐sectional Quantitative analysis Level 6	267 patients with diabetic foot ulcer	Reside in rural areas, lack of regular exercise, peripheral neuropathy	Clinical Socioeconomic
Rosenburg et al. (2023) Sweden *International Wound Journal*	Experiences of undergoing venous leg ulcer management	Describe experiences in the treatment of venous ulcers	Cross‐sectional Qualitative analysis Level 6	16 people with venous ulcers	Health beliefs, fragility in the bond with the team	Psycho‐emotional Health System
Saghdaoui et al. (2023) UK *International Wound Journal*	Healthcare interventions to aid patient self‐management…	Explore self‐care support interventions	Scoping Review Level 5	7 studies	Incorrect or insufficient knowledge	Cognitive
Pouwer et al. (2024) Denmark *Diabetes/Metabolism Research and Reviews*	The quest for wellness	Discuss barriers to foot self‐care	Narrative review Level 5	—	Lack of shared decision‐making	Cognitive Health system
Rosenburg et al. (2024) Sweden *BMC Geriatrics*	Associations between self‐care advice and healing time	Analyse associations between orientations and healing	Cross‐sectional Quantitative analysis Level 6	699 people with chronic wounds	Pain, comorbidities	Clinical

*Note:* Level 1: Systematic reviews and meta‐analyses of randomised controlled trials (RCTs) and Clinical guidelines based on systematic reviews of RCTs; Level 2: RCTs; Level 3: Non‐randomised controlled clinical studies and quasi‐experimental studies; Level 4: Cohort and case–control studies; Level 5: Systematic reviews of descriptive and qualitative studies; Level 6: Cross‐sectional studies, qualitative studies and case series.; Level 7: Expert opinion, expert committee reports, and experience reports.

*Source:* Prepared by the authors.

Most of the included studies explicitly referred to the term self‐care (Jones et al. 2003; Gonzalez et al. 2010; Vedhara et al. 2014; Abu‐Qamar 2014; Silva et al. 2016; Garcia et al. 2018; Mohammad and Khresheh 2018; Phillips et al. 2018; Kapp et al. 2018; Lacerda et al. 2019; Fayfman et al. 2020; Negash et al. 2022; Rosenburg et al. 2023, 2024; Pouwer et al. 2024). However, only one study presented a clear definition of self‐care aligned with the World Health Organization's definition, describing self‐care as the capacity of individuals, families and communities to promote and maintain health, prevent disease and cope with illness and disability, with or without the support of a health professional (Rosenburg et al. 2023).

Negash et al. (2022) described self‐care for diabetic foot as care provided by patients for their feet and overall well‐being. Another study (Garcia et al. 2018) conceptualised self‐care as the individual's capacity to care for themselves by performing activities for their own benefit, requiring ongoing commitment to maintain life, health, and well‐being. Zulec et al. (2019) used the term self‐treatment as a synonym for self‐care.

Overall, self‐care was described as a specific act or set of actions aimed at health promotion and disease management. The terms self‐care and self‐management were used interchangeably in several studies (Chin et al. 2019; Costa et al. 2020, 2021; Balet et al. 2021; Saghdaoui et al. 2023), both referring to the active participation of individuals in their treatment.

Evidence indicates that education and knowledge are precursors of self‐care behaviours (Vedhara et al. 2014; Abu‐Qamar 2014). Saghdaoui et al. (2023) defined self‐management as active participation in treatment, comprising three main components: medical, behavioural and emotional management.

Key self‐care activities related to wound management included compression therapy, rest and limb elevation, weight loss and smoking cessation (Silva et al. 2016; Balet et al. 2021; Rosenburg et al. 2024), as well as wound cleansing at each dressing change, application of dressings, foot inspection, use of appropriate footwear and stockings, and attendance at follow‐up appointments (Abu‐Qamar 2014; Chin et al. 2019; Fayfman et al. 2020).

The term self‐care deficit was not explicitly defined in most included studies. However, it was understood as the absence of skills, motivation or active participation in maintaining self‐care practices necessary for recovery from illness or injury and for coping with their consequences (Hernández et al. 2019; Neves et al. 2021).

Given that people living with chronic wounds experience multiple conditions that influence engagement in self‐care practices, the included studies were coded and synthesised into analytical categories, as described below.

### 5.3 Categories of Self‐Care Deficits in People With Chronic Wounds

This review identified four categories derived from the synthesis of the ten conditioning factors described by Dorothea Orem (1995), representing the range of determinants that contribute to ineffective or incomplete self‐care.

#### 5.3.1 Self‐Care Deficits Related to Clinical Factors

Pain, discomfort and fatigue were consistently associated with impaired performance of daily activities and limitations in self‐care (Jones et al. 2003; Hopkins et al. 2006; Kapp et al. 2018; Lacerda et al. 2019; Zulec et al. 2019; Balet et al. 2021; Rosenburg et al. 2023, 2024). Ageing and living with chronic conditions such as obesity, glaucoma, arthritis, cardiovascular disease and diabetes (Jones et al. 2003; Vedhara et al. 2014; Costa et al. 2020; Balet et al. 2021) were also identified as limiting factors that hinder self‐care.

Wound related characteristics, including lesion size and depth, vascular occlusion, tissue hypoxia, local infection, pruritus, odour and excessive exudate (Vedhara et al. 2014; Phillips et al. 2018; Lacerda et al. 2019; Zulec et al. 2019), further influenced engagement in self‐care practices. Collectively, these findings indicate that clinical deficits extend beyond physiological impairment, acting as functional limitations that progressively compromise autonomy and the sustained performance of self‐care activities.

#### 5.3.2 Self‐Care Deficits Related to Socioeconomic Factors

Low socioeconomic status, often reflected in living standards, household income, limited access to health resources or dependence on public healthcare services (Lacerda et al. 2019; Zulec et al. 2019; Costa et al. 2020; Fayfman et al. 2020; Balet et al. 2021; Negash et al. 2022), substantially affects the ability to maintain adequate nutrition, obtain wound care materials, access specialised care and secure appropriate housing conditions. These constraints reduce individuals' capacity to engage in self‐care.

Socioeconomic restrictions limited not only access to material resources but also the continuity of self‐care behaviours, reinforcing inequities in outcomes related to chronic wound management.

#### 5.3.3 Self‐Care Deficits Related to Behavioural, Psycho‐Emotional and Cognitive Factors

Multiple behavioural, psycho‐emotional and knowledge‐related factors were identified as determinants of self‐care deficits, including symptom‐related beliefs, substance misuse, depression, fear, shame, stress, emotional distress and self‐blame (Jones et al. 2003; Gonzalez et al. 2010; Vedhara et al. 2014; Garcia et al. 2018; Phillips et al. 2018; Lacerda et al. 2019; Fayfman et al. 2020; Negash et al. 2022; Rosenburg et al. 2023; Pouwer et al. 2024).

Insufficient knowledge about the condition, cognitive impairments or lack of clear and accessible guidance reduced understanding and execution of self‐care measures, negatively affecting treatment adherence (Abu‐Qamar 2014; Vedhara et al. 2014; Phillips et al. 2018; Mohammad and Khresheh 2018; Zulec et al. 2019; Costa et al. 2020; Balet et al. 2021; Saghdaoui et al. 2023). Limited knowledge about the disease, rest and dietary choices, as well as beliefs rooted in common sense, contributed to the avoidance of certain foods (e.g., pork, chocolate, mango, okra and cabbage) and failure to monitor glycaemic levels (Silva et al. 2016; Chin et al. 2019; Costa et al. 2021).

These findings highlight that self‐care deficits are frequently mediated by cognitive and emotional processes, in which misinformation, emotional distress and dysfunctional beliefs directly compromise adherence and self‐care capacity.

#### 5.3.4 Self‐Care Deficits Related to Health System Factors

Several studies reported issues related to the availability, accessibility and quality of healthcare services (Garcia et al. 2018; Kapp et al. 2018; Chin et al. 2019; Zulec et al. 2019; Costa et al. 2020, 2021; Fayfman et al. 2020; Rosenburg et al. 2023, 2024; Pouwer et al. 2024). Determinants within this category included limited access to services and resources, long waiting times, difficulties scheduling appointments, dissatisfaction with treatment outcomes, lack of trust in healthcare professionals, communication deficits and absence of shared decision‐making.

Health system barriers emerged as structural determinants of self‐care deficits by restricting access to care, weakening therapeutic relationships and limiting shared decision‐making processes.

## 6 Discussion

The findings of this review demonstrate that self‐care is a central component in the management of chronic wounds and represents a dynamic process influenced by multiple determinants. Caring for oneself involves awareness of one's health status and depends on internal and external motivations, learned behaviours, cultural norms, life contexts and the social environment (Hernández et al. 2019). Thus, self‐care cannot be understood as an isolated or exclusively individual practice.

In the literature, self‐care is conceptualised in heterogeneous ways. The World Health Organization (WHO 2024) defines self‐care as “the ability of individuals, families and communities to promote and maintain health, prevent disease and cope with illness, with or without the support of a health or care professional.” Orem conceptualises self‐care as “the practice of activities initiated and performed by individuals on their own behalf for the maintenance of life, health and well‐being” (Orem 1971), which is the theoretical framework adopted in this study.

In several of the studies reviewed, the terms *self‐care* and *self‐management* are used interchangeably. However, self‐care represents a broader construct that encompasses a wide range of behaviours aimed at the promotion and maintenance of health. In contrast, self‐management, within the context of chronic conditions, predominantly refers to formally prescribed activities, such as medication use, self‐monitoring, and therapeutic management supported by technology, and can therefore be understood as a subcategory of self‐care (Martínez et al. 2021; Ferguson et al. 2024). The conceptual heterogeneity observed across the included studies highlights the need for greater theoretical and conceptual rigour in both research and clinical practice.

From this multidimensional perspective, and based on the categories identified in this review—considered individually or in combination—self‐care deficits arise from the interaction of clinical, socioeconomic, behavioural, psycho‐emotional, cognitive and structural determinants related to health systems. This understanding legitimises the role of nursing in identifying self‐care deficits, systematically assessing individuals' capacities and limitations, establishing diagnoses, applying validated instruments and, based on this assessment, guiding interventions that enhance autonomy and improve clinical outcomes (Yip 2021; Changsieng et al. 2023).

Determinants such as level of knowledge (Saghdaoui et al. 2023), the influence of underlying clinical conditions, and access to and responsiveness of health systems (Lacerda et al. 2019; Vedhara et al. 2014; Costa et al. 2020) emerged as particularly relevant variables for the occurrence of self‐care deficits.

With regard to clinical determinants, conditions such as pain, discomfort, fatigue and chronic diseases—including diabetes and cardiovascular disease—are frequently associated with reduced functional capacity and limitations in activities of daily living, directly affecting self‐care practices (Lacerda et al. 2019; Vedhara et al. 2014). In addition, specific wound characteristics, such as size, depth, infection, excessive exudate, unpleasant odour and pruritus, directly influence patients' engagement in wound care management (Phillips et al. 2018; Zulec et al. 2019).

Hyperglycaemia associated with chronic inflammation, circulatory dysfunction, hypoxia and neuropathy disrupts biological healing processes (Eleftheriadou et al. 2021). Hypertension predisposes individuals to cellular, molecular and haemodynamic changes in the microcirculatory system through activation of inflammatory cascades and proteolytic activity, leading to structural alterations in capillary vessels, tissue hypoxia and delayed angiogenesis during the proliferative phase of wound healing (Raffetto et al. 2020).

This constellation of conditions hinders the performance of recommended practices, such as wound cleansing and dressing changes, and generates frustration and demotivation among patients, as highlighted by Hopkins et al. (2006). Recent studies further emphasise the importance of understanding the determinants of self‐care deficits experienced by individuals managing chronic wounds (Goh and Zhu 2024; Zhang et al. 2025; Zhao et al. 2025). Research by Woo et al. (2024) indicates that persistent pain, associated with discomfort and difficulty walking, is among the most frequently reported patient complaints. These factors limit the performance of basic self‐care practices and negatively affect quality of life, leading to social isolation and increased dependence on caregivers.

Socioeconomic determinants were also strongly associated with self‐care deficits. Poverty, inadequate housing, low income, lack of basic hygiene conditions, and dependence on public health systems exacerbate the challenges faced by people living with chronic wounds (Lacerda et al. 2019). These conditions may reduce the frequency with which individuals seek care and perform necessary self‐care practices, such as maintaining a healthy diet, purchasing wound care supplies, and accessing specialised services (Gethin et al. 2020; Slavich et al. 2023).

Nutritional deficiencies, resulting from both resource scarcity and cultural beliefs, significantly impair wound healing, particularly deficiencies in protein, vitamins A, C and E, and certain micronutrients. Depending on age, comorbidities, body weight and lifestyle habits, wound healing may require an energy expenditure of up to 35–40 kcal/kg per day (Ghaly et al. 2021). Moreover, common‐sense beliefs and culturally shaped dietary practices interfere with adherence to nutritional guidance and self‐care behaviours (Silva et al. 2016; Vedhara et al. 2014).

Slavich et al. (2023) highlight that socioenvironmental factors modulate tissue regeneration mechanisms through regulation of neural and immune system components involved in inflammation. Various determinants—such as low income, low literacy levels, sociofamily conflicts, isolation, rejection and exclusion—may trigger psychological and biological changes leading to chronic systemic inflammation, partly mediated by epigenetic alterations in immune responses (Hajj et al. 2024).

From a psychosocial perspective, conditions such as depression, fear, stress, and shame are common among people with chronic wounds and are associated with neglect of care practices (Gonzalez et al. 2010; Pouwer et al. 2024). Evidence indicates that psychosocial stress activates the hypothalamic–pituitary–adrenal axis, increasing glucocorticoid and catecholamine release, altering immune responses, and consequently impairing wound repair (Fayne et al. 2020; Basu et al. 2024).

Barriers related to health systems emerged as relevant structural determinants. Difficulties in access, long waiting times, communication failures, lack of continuity of care and absence of shared decision‐making weaken therapeutic relationships and reduce patient engagement (Zulec et al. 2019; Costa et al. 2020; Rosenburg et al. 2023; Chin et al. 2019). A recent review corroborates these findings, identifying structural limitations, workforce overload, resource shortages and weak interdisciplinary collaboration as factors compromising care for people with chronic wounds (Mohr et al. 2024).

In line with the WHO framework, clinical practice should adopt a person‐centred, integrated and continuous approach, recognising people living with chronic wounds as active participants in the care process (World Health Organization 2024). From the perspective of Orem's Self‐Care Deficit Theory, the findings highlight the presence of self‐care deficits related to both therapeutic demands and individual and contextual limitations. In this context, nursing plays a central role in identifying these deficits and implementing supportive–educative systems that promote the progressive development of self‐care capacity (Callender et al. 2021).

At the policy level, there is a clear need to incorporate principles of integrated, longitudinal and person‐centred care, articulating different levels of healthcare delivery, with emphasis on early intervention, multidisciplinary management and the adoption of emerging technologies to ensure sustainable and equitable care models (Probst et al. 2025; Sen 2025).

## 7 Limitations

This scoping review provides a comprehensive synthesis of the determinants of self‐care deficits in people living with chronic wounds; nevertheless, some limitations should be acknowledged. The included studies exhibited considerable methodological heterogeneity in terms of study design, sample size, and populations investigated, which may have influenced the findings and limited the generalisability of the results. In addition, although the concept of self‐care was widely addressed in the literature, its operationalisation varied substantially, and the concept of self‐care deficit was often described indirectly or inconsistently. This conceptual variability may hinder comparisons across studies and limit the development of targeted interventions.

These limitations highlight the need for further research, including co‐produced reviews, longitudinal studies with active public involvement, investigations focusing on vulnerable populations, and studies evaluating the impact of tailored interventions. Further research is also required to explore the use of technologies and their potential to support self‐care and enable early identification of self‐care deficits, particularly among hard‐to‐reach populations.

## 8 Conclusion

This scoping review demonstrates that self‐care deficits in people living with chronic wounds constitute a multifactorial and dynamic phenomenon resulting from the interaction of clinical, socioeconomic, behavioural, and health system–related determinants. Clinical conditions such as pain, comorbidities, and functional limitations, combined with socioeconomic inequalities, low educational attainment, and insufficient social support, compromise individuals' capacity and opportunities for self‐care. In addition, barriers within health systems and behavioural factors, including knowledge gaps, maladaptive beliefs, low self‐efficacy, and difficulties with treatment adherence, further increase vulnerability throughout the disease trajectory.

The findings reinforce that a lack of recognition or consideration of these determinants weakens care delivery and contributes to predominantly technical, procedure‐focused practices. World Health Organization frameworks and nursing theories, such as Orem's Self‐Care Theory, support a comprehensive understanding of the multiplicity of these factors and underscore the need for integrated approaches grounded in theoretical models of self‐care. Such approaches should incorporate personalised educational strategies to empower individuals, strengthen autonomy, promote behaviour change and guide clinical interventions, public health policies and future research aimed at improving outcomes and quality of life for people living with chronic wounds.

Within this context, nursing plays a fundamental role, particularly when evidence‐based practice is combined with theoretical frameworks in professional practice. This integration promotes a holistic, person‐centred approach that considers individuals' needs within their life contexts and contributes to empowerment in health care.

## Author Contributions

All the authors are accountable for the preparation of this manuscript. Specifically, Janislei Soares Dantas designed the review and registered the protocol. Janislei Soares Dantas, Maria Eliane Moreira Freire and Selene Cordeiro Vasconcelos designed the research questions and contributed to the study design. Yan Lincoln Mamede Gomes and Janislei Soares Dantas developed, refined, applied the search strategies, screened, selected the studies and performed data extraction. Maria Eliane Moreira Freire contributed third reviewer when needed. Janislei Soares Dantas and Ana Clara de Macedo Farias Ramos prepared and drafted the manuscript. Maria Eliane Moreira Freire and Selene Cordeiro Vasconcelos reviewed the manuscript. All authors have revised the manuscript. Ana Gomes formatted according to the journal's guidelines. All the authors have read the final version of the manuscript and given final approval for the version to be published. Each author participated sufficiently in the work to take public responsibility for appropriate for the content.

## Funding

The authors have nothing to report.

## Disclosure

Protocol Registration: Open Science Framework (https://doi.org/10.17605/OSF.IO/EC9WZ).

## Ethics Statement

The authors have nothing to report.

## Consent

The authors have nothing to report.

## Conflicts of Interest

The authors declare no conflicts of interest.

## Data Availability

The search strategies (Appendix 1) has been incorporated into the main manuscript. The other data that supports the findings of this study are available from the corresponding author upon reasonable request.

## Appendix 1 Search Strategy

**Table:**

Databases	Search strategy	*N*
Web of Science	(((TI = ((chronic wound*) OR ulcer* OR injur* OR wound healing OR wound care)) AND AB = ((venous OR varicose OR stasis OR arter* OR leg OR hard‐to‐heal OR extremity OR hypertens* OR mixed OR diabet* OR neuropath* OR foot OR plantar OR decubitus OR bed* OR ischemi* OR neuroischemi* OR ischaemi* OR neuroischaemi* malignan OR pressure))) NOT AB = ((respiratory OR pulmonary OR renal OR kidney OR hepat* OR lung OR pancrea OR bowel OR colorect* OR liver OR cancer OR tumo* OR spinal OR bone OR brain OR muscle OR eye OR retinal OR tract OR dental OR vertebral OR urethal OR bladder OR appendi* OR gastric OR gastrointestinal OR colorectal OR cataract OR corneal OR gastrectomy OR oesophageal Ooesophageal OR mounth OR stress OR genital oral OR cartilage OR burns))) AND AB = ((Self Care) OR Self‐Management OR Self‐Treatment OR Patient participation)	183
Medline (PubMed)	((((“chronic wound*”[Title/Abstract]) OR “ulcer*”[Title/Abstract] OR “injur*”[Title/Abstract] OR “wound healing”[Title/Abstract] OR “wound care”[Title/Abstract]) AND ((“venous”[Title/Abstract] OR “varicose”[Title/Abstract] OR “stasis”[Title/Abstract] OR “arter*”[Title/Abstract] OR “leg”[Title/Abstract] OR “hard‐to‐heal”[Title/Abstract] OR “extremity”[Title/Abstract] OR “hypertens*”[Title/Abstract] OR “mixed”[Title/Abstract] OR “diabet*”[Title/Abstract] OR “neuropath*”[Title/Abstract] OR “foot”[Title/Abstract] OR “plantar”[Title/Abstract] OR “decubitus”[Title/Abstract] OR “bed*”[Title/Abstract] OR “ischemi*”[Title/Abstract] OR “neuroischemi*”[Title/Abstract] OR “ischaemi*”[Title/Abstract] OR’ “malignan”[Title/Abstract] OR “pressure”[Title/Abstract]))) NOT ((“respiratory”[Title/Abstract] OR “pulmonary”[Title/Abstract] OR “renal”[Title/Abstract] OR “kidney”[Title/Abstract] OR “hepat*”[Title/Abstract] OR “lung”[Title/Abstract] OR “pancrea”[Title/Abstract] OR “bowel”[Title/Abstract] OR “colorect*”[Title/Abstract] OR “liver”[Title/Abstract] OR “cancer”[Title/Abstract] OR “tumo*”[Title/Abstract] OR “spinal”[Title/Abstract] OR “bone”[Title/Abstract] OR “brain”[Title/Abstract] OR “muscle”[Title/Abstract] OR “eye”[Title/Abstract] OR “retinal”[Title/Abstract] OR “tract”[Title/Abstract] OR “dental”[Title/Abstract] OR “vertebral”[Title/Abstract] OR “urethal”[Title/Abstract] OR “bladder”[Title/Abstract] OR “appendi*”[Title/Abstract] OR “gastric”[Title/Abstract] OR “gastrointestinal”[Title/Abstract] OR “colorectal”[Title/Abstract] OR “cataract”[Title/Abstract] OR “corneal”[Title/Abstract] OR “gastrectomy”[Title/Abstract] OR “oesophageal Ooesophageal”[Title/Abstract] OR “mounth”[Title/Abstract] OR “stress”[Title/Abstract] OR “genital oral”[Title/Abstract] OR “cartilage”[Title/Abstract] OR “burns”[Title/Abstract]))) AND ((“Self Care”[Title/Abstract]) OR “Self‐Management”[Title/Abstract] OR “Self‐Treatment”[Title/Abstract])	75
Cochrane Library	(chronic wound*) OR ulcer* OR injur* OR wound healing OR wound care in Abstract AND (venous OR varicose OR stasis OR arter* OR leg OR hard‐to‐heal OR extremity OR hypertens* OR mixed OR diabet* OR neuropath* OR foot OR plantar OR decubitus OR bed* OR ischemi* OR neuroischemi* OR ischaemi* OR neuroischaemi* malignan OR pressure) in Abstract NOT (respiratory OR pulmonary OR renal OR kidney OR hepat* OR lung OR pancrea OR bowel OR colorect* OR liver OR cancer OR tumo* OR spinal OR bone OR brain OR muscle OR eye OR retinal OR tract OR dental OR vertebral OR urethal OR bladder OR appendi* OR gastric OR gastrointestinal OR colorectal OR cataract OR corneal OR gastrectomy OR oesophageal Ooesophageal OR mounth OR stress OR genital oral OR cartilage OR burns) in Abstract AND (Self Care) OR Self‐Management OR Self‐Treatment in Abstract—(Word variations have been searched)	7
Scopus (Elsevier)	(ABS ((chronic AND wound*) OR ulcer* OR injur* OR wound AND healing OR wound AND care) AND ABS ((venous OR varicose OR stasis OR arter* OR leg OR hard‐to‐heal OR extremity OR hypertens* OR mixed OR diabet* OR neuropath* OR foot OR plantar OR decubitus OR bed* OR ischemi* OR neuroischemi* OR ischaemi* OR neuroischaemi* AND malignan OR pressure)) AND NOT ABS ((respiratory OR pulmonary OR renal OR kidney OR hepat* OR lung OR pancrea OR bowel OR colorect* OR liver OR cancer OR tumo* OR spinal OR bone OR brain OR muscle OR eye OR retinal OR tract OR dental OR vertebral OR urethal OR bladder OR appendi* OR gastric OR gastrointestinal OR colorectal OR cataract OR corneal OR gastrectomy OR oesophageal AND ooesophageal OR mounth OR stress OR genital AND oral OR cartilage OR burns)) AND ABS (self‐care OR self‐management OR self‐treatment)) AND PUBYEAR > 2013 AND PUBYEAR < 2025	996
Scielo.Org	(ab:(*(chronic wound*) OR ulcer* OR injur* OR wound healing OR wound care)) AND (ab:((venous OR varicose OR stasis OR arter* OR leg OR hard‐to‐heal OR extremity OR hypertens* OR mixed OR diabet* OR neuropath* OR foot OR plantar OR decubitus OR bed* OR ischemi* OR neuroischemi* OR ischaemi* OR neuroischaemi* malignan OR pressure))) AND NOT (ab:((respiratory OR pulmonary OR renal OR kidney OR hepat* OR lung OR pancrea OR bowel OR colorect* OR liver OR cancer OR tumo* OR spinal OR bone OR brain OR muscle OR eye OR retinal OR tract OR dental OR vertebral OR urethal OR bladder OR appendi* OR gastric OR gastrointestinal OR colorectal OR cataract OR corneal OR gastrectomy OR oesophageal Ooesophageal OR mounth OR stress OR genital oral OR cartilage OR burns))) AND (ab:((Self Care) OR Self‐Management OR Self‐Treatment))	975
Ovid (Journals Full Text)	((((chronic wound* or ulcer* or injur* or wound healing or wound care) and (venous or varicose or stasis or arter* or leg or hard‐to‐heal or extremity or hypertens* or mixed or diabet* or neuropath* or foot or plantar or decubitus or bed* or ischemi* or neuroischemi* or ischaemi* or neuroischaemi* malignan or pressure)) not (respiratory or pulmonary or renal or kidney or hepat* or lung or pancrea or bowel or colorect* or liver or cancer or tumo* or spinal or bone or brain or muscle or eye or retinal or tract or dental or vertebral or urethal or bladder or appendi* or gastric or gastrointestinal or colorectal or cataract or corneal or gastrectomy or oesophageal Ooesophageal or mounth or stress or genital oral or cartilage or burns)) and (Self Care or Self‐Management or Self‐Treatment)).ab	9
EMBASE (Elsevier)	(chronic AND wound* OR ulcer* OR injur* OR ‘wound healing’/exp. OR ‘wound healing’ OR ((‘wound’/exp. OR wound) AND (‘healing’/exp. OR healing)) OR ‘wound care’/exp. OR ‘wound care’ OR ((‘wound’/exp. OR wound) AND (‘care’/exp. OR care))) AND (venous:ab,ti OR varicose:ab,ti OR stasis:ab,ti OR arter*:ab,ti OR leg:ab,ti OR ‘hard to heal’:ab,ti OR extremity:ab,ti OR hypertens*:ab,ti OR mixed:ab,ti OR diabet*:ab,ti OR neuropath*:ab,ti OR foot:ab,ti OR plantar:ab,ti OR decubitus:ab,ti OR bed*:ab,ti OR ischemi*:ab,ti OR neuroischemi*:ab,ti OR ischaemi*:ab,ti OR ‘neuroischaemi* malignan’:ab,ti OR pressure:ab,ti) NOT (respiratory:ab,ti OR pulmonary:ab,ti OR renal:ab,ti OR kidney:ab,ti OR hepat*:ab,ti OR lung:ab,ti OR pancrea:ab,ti OR bowel:ab,ti OR colorect*:ab,ti OR liver:ab,ti OR cancer:ab,ti OR tumo*:ab,ti OR spinal:ab,ti OR bone:ab,ti OR brain:ab,ti OR muscle:ab,ti OR eye:ab,ti OR retinal:ab,ti OR tract:ab,ti OR dental:ab,ti OR vertebral:ab,ti OR urethal:ab,ti OR bladder:ab,ti OR appendi*:ab,ti OR gastric:ab,ti OR gastrointestinal:ab,ti OR colorectal:ab,ti OR cataract:ab,ti OR corneal:ab,ti OR gastrectomy:ab,ti OR ‘oesophageal ooesophageal’:ab,ti OR mounth:ab,ti OR stress:ab,ti OR ‘genital oral’:ab,ti OR cartilage:ab,ti OR burns:ab,ti) AND (‘self‐care’:ab,ti OR ‘self‐management’:ab,ti OR ‘self‐treatment’:ab,ti OR ‘health determinants’:ab)	831
